# Folk classification of the crabs and swimming crabs (Crustacea – Brachyura) of the Mamanguape river estuary, Northeastern – Brazil

**DOI:** 10.1186/1746-4269-5-22

**Published:** 2009-08-11

**Authors:** Emmanoela N Ferreira, José da S Mourão, Pollyana D Rocha, Douglas M Nascimento, Dandara Monalisa Mariz da S Q Bezerra

**Affiliations:** 1 Programa de Pós-Graduação em Ciências Biológicas, Zoologia, Departamento de Sistemática e Ecologia, Centro de Ciências Exatas e da Natureza, Universidade Federal da Paraíba, Campus 1, Cidade Universitária, P.O. 58059-900, João Pessoa, PB, Brazil; 2 Departamento de Biologia, Universidade Estadual da Paraíba, Av, Baraúnas, nr. 351/Campos Universitário, Bodocongó, P.O. 58109-753, Campina Grande, PB, Brazil; 3 Curso de Licenciatura e Bacharelado em Ciências Biológicas, Universidade Estadual da Paraíba, Av, Baraúnas, nr. 351/Campos Universitário, Bodocongó, P.O. 58109-753, Campina Grande, PB, Brazil

## Abstract

**Background:**

Folk taxonomy is a sub-area of ethnobiology that study the way of how traditional communities classify, identify and name their natural resources. The work present was undertaken in two traditional communities (Barra de Mamanguape and Tramataia). The objective of this study was investigate the ethnobiological classification of the local crabs and swimming crabs used by the crustaceous gatherers of the Mamanguape River Estuary (MRE), Paraíba State, Brazil.

**Methods:**

The methodology used here involved a combination of qualitative methods (open interviews, semi-structured interviews, direct observations, guided tours, surveys, and interviews in synchronic and diachronic situations that crossed-checked and repeated identifications) and quantitative methods (Venn diagram). A total of 32 men and women were interviewed in the two communities. Specimens of the local crustaceans were collected and identified by the harvesters themselves, subsequently fixed in formalin, conserved in 70% ethyl alcohol, identified using appropriate specialized literature, and then deposited in the laboratory of the Zoology Department of the University State of Paraiba.

**Results:**

The crustaceous gatherers we studied were observed to group crustaceans according to their similarities and differences, producing a hierarchical classification system containing four levels of decreasing taxonomic order: unique beginner, life-form, generic, and specific. A sequential and/or semantic system classification system that is used to classify the ontogeny of the female swimming crab was also identified.

Of the nine folk generics identified, 44.5% were monotypic. 55.5% were polytypic and were subdivided into 15 folk specifics.

An identification key was elaborated with the data obtained about the folk polytypics generics.

**Conclusion:**

The detailed knowledge concerning the crabs and swimming crabs revealed by the MRE crustaceous gatherers demonstrates that these people detain a vast knowledge concerning these marine resources. This local knowledge provides a rich but little-known source of information that will aid future ecological and/or zoological studies in the region that will be indispensable for producing management plans to help guarantee the sustainability of these local natural resources.

## Background

Traditional (local) communities retain detailed knowledge about the biological resources of their surrounding environment. The experiences, knowledge, and knowing-doing accumulated by these communities about the natural and supernatural worlds and which is transmitted orally through the generations, characterizes traditional or local knowledge [1-4].

Studies concerning traditional knowledge are themes of the ethnosciences – a branch of study that arose as a fusion of fields and that has continued to evolve in the exchange between the natural sciences and the human/social sciences [5]. Ethnoscience grows with each day, but it also tends to fragment into sub-areas (like most of modern science) – in contrast to the communities under study, which don't tend to fragment their knowledge. As such, ethnobiology is a sub-area of the ethnosciences that seeks to understand and analyze the way in which living things are perceived, known, and classified by diverse human communities [6,7]. Research directed towards the various areas of the ethnosciences (ethnobotany, ethnoichthyology, ethnobiology) have come to age in the scientific world and have contributed to recent investigations into the knowledge of traditional populations [2].

Ethnobiology can be still further subdivided into folk taxonomy or ethnotaxonomy – which studies how traditional communities classify, identify, and name their natural resources. According to Lévi-Strauss [8], human populations have an apparent intellectual necessity to classify the natural world because it is inherent in humans to demand order. Humans respond to plant and animal diversity in their environment by grouping these living organisms into named categories that express differences and similarities between them, and also group them into classificatory categories of greater or lesser inclusion [9,10].

One of the authors that most stands out in terms of studies of folk taxonomy is Brent Berlin [11] who developed twelve general principals of ethno-biological classification and naming – of which seven are directed towards classification and five towards naming. Among the basic principals proposed by Berlin [11], those that refer to structural hierarchies especially draws our attention – with plants and animals being ordered in a way that establishes hierarchies comparable to those of Linnaean taxonomy [3], with hierarchical categories organized on the basis of principals of inclusion and exclusion, strictly separated and included in more general categories [12]. Many studies throughout the world have demonstrated that the folk classification of animals and plants consist, quite often, to the scientific classification of those organisms and this coherence demonstrates that these classifications are not arbitrary cultural workmanships, but are determined by some degree of biological reality or universal cognition [13]. According to Berlin [14], ethnobiological classifications start with the principal of universality among the different cultures, where there are consistencies in the classification and naming of plants and animals among traditional populations.

Studies of folk taxonomy allow the interaction of the ethnobiological knowledge of traditional populations with more formal scientific knowledge and seek to better understand the diversity of these communities and their relationship with their natural environment [7]. Similar to scientific taxonomy, ethnotaxonomy retains a vast store of information about biology, ecology, and ethology of both animals and plants [15]. The ethnobiological classification system can be used as a tool for a rapid assessment of biodiversity [16], can also contribute to a great deal of new information about the natural resources and even assist in new taxonomic discoveries. Folk taxonomy not only organizes and condenses biological information but it also provides a powerful systematic tool to examine the distribution of biological and ecological properties among organisms [17].

Also according to Castro [18], the classificatory systems of "traditional" populations make up part of their cultural patrimony and their relations to the natural world are manifested in their vocabularies and in the terms the use to translate their experiences and adaptations to the environment around them. As such, ethnotaxonomic studies are important in that they help preserve and conserve the biological and cultural diversity that is reflected in the traditional (usually oral) knowledge of these communities It is important to understand the local diversity in order to get an efficient conservation and management of the resources

The present work examined the folk taxonomy of the crustaceous gatherers of the communities of Barra de Mamanguape and Tramataia on the Mamanguape River Estuary (MRE), Paraíba State, Brazil, in which we analyzed the classification criteria used for the crabs and swimming crabs of that region.

As most of the crustaceous gatherers and fishermen of the region still dedicate most of their lives to manual collecting and fishing, they retain an enormous empirical knowledge about the resources of the region. This traditional knowledge should be respected and consulted when management activities are planned for a given region, and ethnobiological and ethnoecological studies can aggregate the value of their local knowledge and culture to more formal scientific endeavors.

## Methods

### The study area

**Figure 1:**
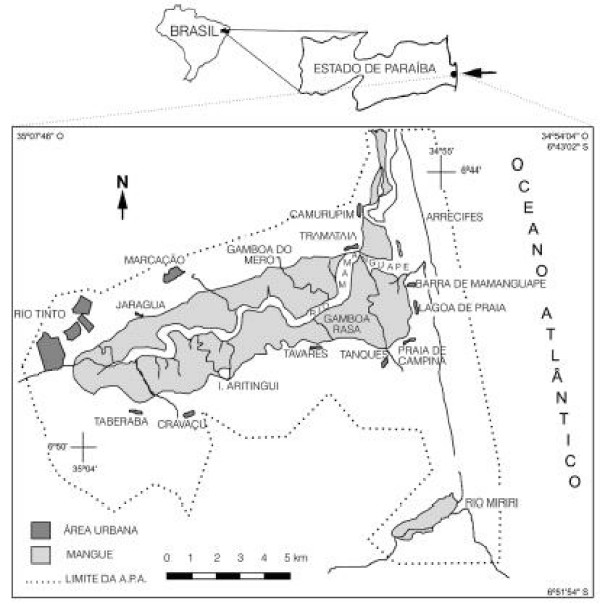
**Map of the localization of the Mamanguape River Estuary – MRE**.

The MRE (Figure 1) is located approximately 80 km from the state capital of João Pessoa (6°43'02" to 6°51'54"S × 35°67'46" to 34°54'04"W) and is considered the second largest estuary in Paraíba State, Brazil [19]. This single mangrove swamp occupies approximately 5400 ha, being larger than all of the other mangrove swamps in that state taken together (10,080 ha. total) [20]. The mangrove swamp is located in an Environmental Protection Area ("EPA") in the northern region of Paraíba State, within the municipalities of Rio Tinto and Marcação.

This mangrove swamp is in a reasonably good state of conservation, although subject to some deforestation by the neighboring populations, continuous silting of the river, occasional contamination by agro-chemicals, and recent devastation due to sugarcane cultivation projects mounted by sugarcane agro-industries [19-22]. The estuary is associated with coastal reefs, tidal lakes, "croas" (sand/mud banks), "apicuns" (areas without the typical mangrove vegetation), and Atlantic Forest areas.

The mangrove swamp in question shows relatively low diversity in terms of its floristic composition, being mostly populated by the species: *Rhizophora mangle *("mangue vermelho" or "sapateiro"), *Avicennia schaweriana *("mangue canoe"), *Avicennia germinans, Laguncularia racemosa *("mangue branco" or "manso"), and *Conocarpus erectus *[19,21,22]. The swamp fauna includes fishes, crustaceans, bivalves mollusks and gastropods, which are the principal subsistence items of the local communities [22].

The present research was undertaken in association with the communities of Barra de Mamanguape and Tramataia that are located in the interior of the EPA and along the margins of the MRE. The villages are composed of a racial mixture of indigenous (Potiguar), black, and white elements that are extremely dependent on the forest and mangrove areas for their subsistence, and they exercise extractivist activities in the areas in and around the Mamanguape river [20,22].

### Procedures

Field work was carried out during the period from January to June, 2007, with visits being made every two weeks. Qualitative methods were used to obtain information about the fishing culture being investigated, emphasizing folk knowledge about the systems used to classify the local crustaceans, and zoological techniques were used for taxonomic surveys.

Data collection was undertaken in two stages. The first stage involved surveys to define and choose local informants, and then open interviews were performed in order to determine the general profile of the target population. The second phase involved open and semi-structured interviews designed to define the domains of the folk crustacean taxonomy. A total of 32 people were interviewed in the two communities (20 men and 12 women) who performed (or used to perform) harvesting activities and/or fishing in the region and who had significant personal knowledge of the subject being studied.

The details of the interviews were recorded manually and/or by using a voice recorder. Transcriptions were performed with full awareness of the need to be faithful to the interviewees, and the tapes are now stored at the Nucleus of Ethnoecology/Ethnobiology of the Universidade Estadual da Paraíba (UEPB). Other techniques were used in addition to the interviews, including direct observations and guided tours – with the objective of integrating the researchers and the interviewees, collect specimens, and experience the reality of these people in their natural environment.

Specimens of the local crustaceans described here were collected and identified in the field by the key-informants themselves. After collection, the specimens were fixed in 10% formalin, conserved in 70% ethanol alcohol, and subsequently deposited into the didactic collection of the Laboratory of Zoology at UEPB.

The scientific identification of the crabs and swimming crabs specimens was undertaken with the use of the appropriate specialized literature [23,24] at the Laboratory of Zoology in the Department of Biology at UEPB, and then compared to the Paulo Young Invertebrate Collections at the Department of Systematics and Ecology of the Universidade Federal da Paraíba (UFPB).

For analysis and control of the data, verification tests were performed to determine the consistency and validity of the responses. This was done by repeating details of the interviews in both synchronic and diachronic situations. A crossed ethnoidentification method was used in which samples identified by certain crustaceous gatherers were subsequently given to others for identification; likewise, a technique of repeated ethnoidentifications was employed in which the same samples that had been identified by the collectors were re-submitted to them after a relatively long period of time (three months) to be identified again. The ethnobiological classification of the crustaceous gatherers presented here is based on the Berlin method [11]. A quantitative method was also used, employing a Venn diagram to visualize comparisons between the folk classification and its corresponding scientific form.

## Results and discussions

### Hierarchical classification

Humans are capable of recognizing, categorizing, and identifying examples of most species, grouping similar species, differentiating them from the others, and transmitting this knowledge to other members of their society [11].

Ethnobiological classification systems can be organized conceptually within a shallow hierarchical structure with six universal hierarchy levels: unique beginner, life-form, intermediate, generic, specific, and varietal [11]. The unique beginner level is only rarely named and is composed of only a single member (vegetal or animal). The life-form hierarchical level includes generics taxa, are few in number (no more than ten or fifteen), are generally polytypics, and the animals or plants belonging to the same taxa normally share the same pattern of habitat and body form. The generic hierarchy is the most representative within ethnobiological classification system and is included among the primary or monotypics names in approximately 80% of all cases. Not all folk systems have all six hierarchical, and four levels are, in fact, most common; the intermediate and varietal levels are rarely encountered. The intermediate level is not normally named, and is designated as a hidden category. Specific taxa are included in the generic taxon, are represented by just a few members, and include organisms that have the greatest cultural importance, and are often grouped among secondary names. When the generic level is subdivided by the specific, this will be the terminal hierarchical level, and the subdivided generic level is denominated polytypic.

Brown [10] refers to life-form as the largest and most heterogeneous group regularly encountered in folk taxonomy (excluding the unique beginner taxon) and cites five of the most common life-forms for animals: fish, bird, snake, wug and mammal. These five life-forms are based on morphological characters, but this same author [10] states that life-forms not so universal or dominant are frequently based on criteria such as habitat, form of locomotion, or their relations with man, among others.

According to Frazão [12], studies of classification systems take two approaches: one emphasizes the symbolic character of the classifications, and is often emphasized by European researchers; the other takes more into consideration studies of biological taxonomic structures and lexical systems, and these studies are usually emphasized by American researchers such as Berlin (who is criticized due to the fact that your studies on the classification of animals and plants are based only on morphology, ignoring their cultural significance). Hunn [25] stated that the purely morphological characters of folk classifications cannot be universally recognized, and this author goes on to criticize the life-form taxon, demonstrating that some life-forms are natural taxa, but that most belong to biologically artificial peripheral taxa. Newmaster et. al. [16] suggests that the mechanisms of ethnobiological classification involve not only morphological and ecological perceptions but also sensorial ones and cultural interests as well.

**Figure 2:**
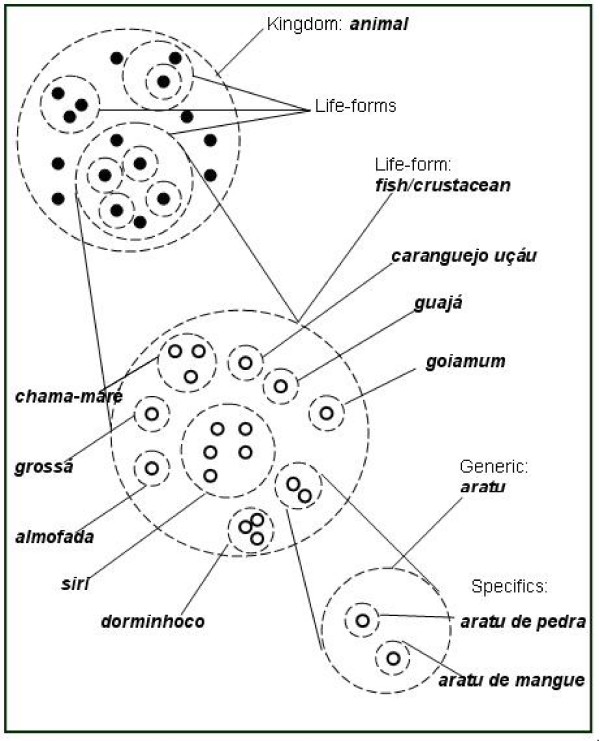
**Ethnobiological classification system and its respective taxa in simultaneous use with the hierarchical classification system**.

The crustaceous gatherers that were interviewed in the present work grouped crabs and swimming crabs based on similarities or differences (morphological and/or ecological) within a hierarchical system compatible with the ethnotaxonomic system described by Berlin, forming, in decreasing order of taxonomic degree, the following levels: unique beginner, life-form, generic, and specific (Figure 2). Jensen [26] encountered a classification system with four hierarchical levels among the Wayãpi Amerindians that he named ethnoclass, ethnofamily, ethnogenus, and ethnospecies. Montenegro [27] recognized that the fishermen of the lower São Francisco River in Brazil grouped shrimp into a hierarchical system with the folk-taxa of generic, life-form, and unique beginner. Costa-Neto and Marques [9] recorded that fishermen of Siribinha (Conde, Bahia State, Brazil) also utilized a hierarchical classification that included the taxa of ethnospecies, ethnofamily, and unique beginner. Paz and Begossi [28] observed a hierarchical classification among the fishermen in Gamboa (Ilha de Itacuruçá, Rio de Janeiro State, Brazil) represented by the folk taxa of life-form, ethnofamily, and ethnospecies. Carrara [29] identified eight hierarchical levels in the Xavante Amerindian classification of birds (also in Brazil). Anderson [30], in studies of the ethnoichthyology of the Cantoneses population in Hong Kong reported a hierarchical classification with only three levels, corresponding to life-form, generic, and specific.

In the present study, hierarchical classifications were implicit in expressions such as: "*there are many types of ****siri***", "*there are two types of ****aratu***", "*there are various types of ****chama-maré***, *one has a large foot, which we call the ****tesoureiro***". Marques [31] observed a similar type of hierarchical classification among the fishermen from the state of Alagoas, with statements such as: "*there are many types*", "*there is a lot of diversity*". Fishermen of Siribinha sub-categorize fish with expressions such as: "*it's like the other*", "*it's in the same family*", and "*it's the same thing*" [32]. Similar expressions of hierarchical categorizing were observed in other studies of folk taxonomy [6,9,12,27,31].

According to a majority of the interviewees at the MRE, crustaceans are considered to be fish, *"it's a fish because it lives in the water, just like fish live in the water"*. These animals share the same habitat, and thus are classified as belonging to the same hierarchical level of life-form. This information, however, is not shared by all members of that society as, for example: *"they're not fish because they have a shell and don't have scales, they're crustaceans"*. The fact that some of the crustaceous gatherers don't consider crustaceans to be fish, however, may be the result of the recent presence of researchers in the region. This perception of the crustaceous gatherers in the MRE is, to a certain extent, corroborated by other studies in folk taxonomy, as for example the work by de Marques [31], who considered the category "fish" very elastic, with the fishermen from Alagoas State including "porpoises, whales, and caimans". According to this author, under certain circumstances some invertebrates are considered fish, as is the case of mollusks and crustaceans, which are consumed cyclically according to the religious calendar ("the fish that we eat during Holy Week"). Mourão and Nordi [33], in their study of the fishermen at the MRE, observed that aquatic vertebrates (manatees, whales) and invertebrates (shrimp and sometimes crabs) are classified as fish. Clément [34], in research on the folk zoology of the Montagnais encountered a very elastic category for fish that included shrimp, lobsters, crabs, and mollusks. In other work with fishing communities [9,27,28], the inclusion of the category "fish" as a life-form for other aquatic species was similarly observed. All of this biological diversity would be included in a taxon understood to be the animal unique beginner [15], as was observed for the crustaceans of the MRE.

In the community studied, the generic hierarchical category most stood out, with more than nine representatives. The generics polytypics taxa that are subdivided into specifics invariably represent those classes of organisms that are culturally important, and the generics monotypics do not include any taxa of inferior orders. Additionally, the recognition of generic polytypic is the result of the biological diversity present in some regions. As such, the results obtained here demonstrate that the generic polytypic were the most expressive taxa in the classification of crabs and swimming crabs at the MRE, corresponding to 55.5% of the total, while generics monotypics represented 44.5% of the total of generics of the crustaceans at the MRE. The predominance of the generics polytypics here is an exception in folk taxonomy, because, according to Berlin [11], a majority of all of the generics taxa is monotypic.

The generics monotypics are represents by the following crustaceans: ***almofada ***(*Aratus pisonii*), ***caranguejo uçáu ***(*Ucides cordatus*), ***grossá ***(*Ocypode quadrata*) e ***guajá ***(*Calappa ocellata*). The diversity of folk specifics for each of the five generics polytypics can be seen in Table 1, where the folk generic "***siri****" *was the most representative (containing six folk specifics). This generic is of great economic and cultural importance in the region studied – which tends to confirm that the generics polytypics group items of greatest local economic, cultural, and physiological importance [6]. But this is not a fixed rule in the MRE, for although the generic "***caranguejo-uçáu***" is classified as a monotypic member, it also to have great economic and cultural importance.

**Table 1:** Diversity of folk species and their scientific equivalents

**Generics polytypics**	**Folk specifics**	**Species**
***aratu***	***aratu de mangue***	*Goniopsis cruentata*
	
	***aratu de pedra***	*Plagusia depressa*

***chama-maré***	***chama-maré de mangue***	*Uca burgersi*
	
	***chama-maré tesoureiro***	*Uca maracoani*
	
	***chama-maré da beira da praia***	*Uca sp*.

***dorminhoco***	***dorminhoco do mangue***	*Panopeus lacustris*
	
	***dorminhoco do mangue***	*Eurytium limosum*
	
	***dorminhoco das pedras***	*Menippe nodifrons*

***goiamum***	***goiamum caboclo***	*Cardisoma guanhumi*
		
	***goiamum azulão***	

***siri***	***siri pontinha***	*Callinectes danae*
	
	***siri açú***	*Callinectes exasperatus*
	
	***siri cagão(M)***	*Callinectes bocourti*
		
	***siri nema(F)***	
	
	***siri pimenta***	*Callinectes larvatus*
	
	***siri pintado/siri das pedras***	*Arenaeus cribrarius*

Legend: M – male; F – female.

The specifics taxa are quite similar except for a few distinctive morphological characters, many of which are easily seen and sometimes verbally described [11]. The specifics of each generics polytypics encountered in the MRE were very similar, differing only in terms of a few characters, such as coloration, size, shape of the carapace, and the thickness of their chelipeds. The "***goiamum caboclo***" and "***goiamum azulão***", for example, differ in terms of their color, as also occurs with the "***dorminhoco do mangue***" and "***dorminhoco das pedras***". The difference between the "***chama-maré tesoureiro***" and "***chama-maré de mangue***" is limited to the thickness of their chelipeds.

### Sequential and/or semantic classification

According to Marques [31], the folk systematics of the fishermen which he studied had four types of classificatory systems: a hierarchical system, a sequential system, a concentric system, and a cyclical system. This same author goes on to cite another type of classification called an "ecological classification" where the animals are grouped according to the habitat in which they live.

**Figure 3:**
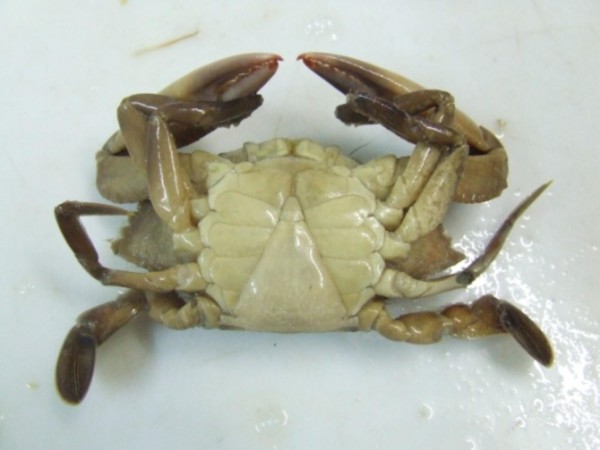
**"*Siri donzela*"/*Callinectes sp*. (Photo: Pollyana Dias, 2007)**.

**Figure 4:**
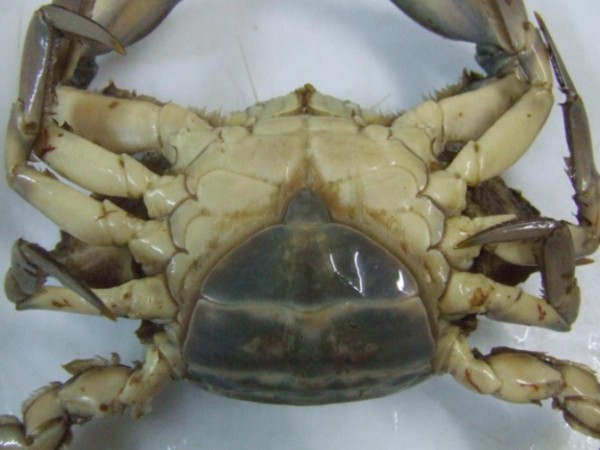
**Abdomen of the *siri *adult female (*Callinectes sp)*. (Photo: Pollyana Dias, 2007)**.

The gatherers of the MRE not only group crustaceans into a hierarchical system, but also use a sequential and/or semantic system to classify the ontogeny of the swimming crabs females, calling those that are considered "virgins" as "***siri donzela***". The "***siri donzela***" is identified by the crustaceous gatherers by the shape of the abdomen (Figure 3), which they describe as being similar to that of the males although it is more triangular while that of the adult female is wider (Figure 4). According to Marques [31], sequential classifications are characterized by a serial ordination according to the morphology and size of the individuals. Williams [24], Almeida [35] and Narchi [36] noted that the sexual maturity of the females of *Callinectus sp*. is judged by the shape of the abdomen – where the abdomens of the young females has a triangular form while those of the adult females are more semicircular. According to Barreto, Batista-Leite and Aguiar [37] the ontogeny of crustaceans is determined by morphological changes caused by the differential growth of certain tagmas.

*"It's because when she is a donzela, she does have a complete carapace – looks like a siri (male), the male siri only cross with her when she molts, then when she molts her carapace grows and widens" *(Hélio, gatherers at the MRE, referring to "*siri donzela*").

*"Because she is young, she is a virgin and (we) call her a donzela. She hasn't yet mated. Her carapace is narrow. After she mates then she will grow that covering" *(Cleonice, gatherers at Tramataia, referring to "*siri donzela*").

This same ontogenetic character has been identified in other ethnobiological works, such as the study by Souto [38] in which sequential ontogeny was observed in fish and shrimp; Mourão [22] who noted different names for small or immature fish; Marques [39] who also reported an ontogenetic character in the feeding regime of "Tubarana" (*Salminus hilarii*); Costa-Neto, Dias and Melo [40] who reported that the fish in their study area were classified by the fishermen by a serial ordination of morphology and size; Costa-Neto and Marques [9] observed ontogenetic phases in their studies of ethno-taxonomy by fishermen in Bahia State; and Montenegro [27] who observed differential denominations for young fishes and shrimps.

### Emic identification key

The principal objective of an identification key is to arrive at the precise taxon of the specimen in question using secure characters and choices. Classification keys have enormous practical importance in facilitating the rapid and correct identification of unknown specimens [41].

There have been very few published works elaborating emic identification keys, as for example that of Mourão and Montenegro [6] for fish at the MRE, in Paraíba State, Brazil. The confection of an emic identification key represents an important methodological tool that can be used as a guide to the "ethnodiversity" to be found in the communities studied [6]. Emic identification keys can also greatly assist future work in ethnobiology, ecology, and zoology, for they allow the researcher to more rapidly identify the folk-species of a given region and even to discover new species. These keys do not eliminate the need to consult published traditional scientific descriptions of the groups identified, nor the eventual comparison of the samples collected/observed with study collections to confirm their identification [41].

The crustaceous gatherers of the MRE were observed to possess a vast knowledge of the morphology of these animals, and the functioning of their various body parts that aids in the identification of the various species, in differentiating between them, distinguishing their sexes, and determining the state of sexual maturity of these crustaceans. In possession of this data concerning the folk taxonomy used by the crustacean harvesters at the MRE, it was possible to elaborate an emic identification key (Appendix 1) for the generic polytypics of the crustaceans found there.

## Conclusion

Two classification systems of crustaceans were identified among the crustaceous gatherers in the MRE a hierarchical classification system and a sequential and/or semantic classification system. The hierarchical classification system was observed to be compatible with the model developed by Berlin, with four hierarchical levels, unique beginner (not denominated), life-form, generic, and specific – which allows its comparison with Linnaean taxonomy. The sequential and/or semantic classification system was identified in the ontogenetic classification of the female swimming crabs. The subdivision of the generics folk polytypics into folk specifics was observed principally among the crustaceans demonstrating the greatest economic and cultural importance. It was further observed that for the nine folk generics cited by the interviewees, five (55.5%) were generics polytypics and four (44.5%) were generics monotypics.

With the data obtained concerning the folk generics it was possible to elaborate an emic identification key to aid in the future identification of those animals.

The totality of the knowledge retained by the crustaceous gatherers in the MRE communities about crabs and local swimming crabs will be of significant importance for future ecological and/or zoological studies in the region and for management plans focusing on local sustainability, requiring that this local knowledge be preserved together with the local biological diversity.

## Competing interests

The authors declare that they have no competing interests.

## Authors' contributions

ENF collected and analyzed the data, realized the discussion, identified some specimens of the crabs and wrote the manuscript. JSM designed the study, conducted the framework, supervised the research, revised the manuscript and assisted in the taxonomic identification of the some specimens. PDR collected the data and realized the register photographic. DMN and DMMSQB collected the data. All authors read and approved the final manuscript.

## Appendix 1

Emic identification key for the crabs and swimming crabs (generic polytypics).

ARATU

"*They are red, the youngest are black and have hairs on their fingers. They're smaller than the "caranguejo uçáu". They have one large and one small claw. They walk on tip-toes. They are more common in the mangrove swamp where they stay in the trees*."............................................................................................ **ARATU DE MANGUE**

...............................................................................................................*Goniopsis cruentata*

"*They have a rounder shell, their fingers are longer than those of the "aratu do mangue". They are squat. All of their feet are the same size. They are gray and white below their chest and their fingers. Larger, meatier, taste like lobster, dark brown like sargasso, mud and live among the rocks, only on the reefs*." ........................... **ARATU DE PEDRA**

...................................................................................................................*Plagusia depressa*

CHAMA-MARÉ

"*They're different than those at the tide's edge, the claw is larger and thinner, they live in the mangrove swamp*."................................................... **CHAMA-MARÉ DE MANGUE**

............................................................................................................................*Uca burgesi*

"Very white or yellow and small, live at the edge of the surf."................................................................ **CHAMA-MARÉ DA BEIRA DA PRAIA**

....................................................................................................................................*Uca sp*.

"*They have a red claw, and live at the edge of the croa (mud banks) and at the edge of the surf where there is mud*."..............................................**CHAMA-MARÉ TESOUREIRO**

.......................................................................................................................*Uca maracoani*

DORMINHOCO

*"They live in the mangrove swamp and are darker."*...**DORMINHOCO DO MANGUE**

................................................................................*Panopeus lacustris/Eurytium limosum*

*"They live among the rocks and are lighter than the "dorminhoco do mangue." *.........................................................................................**DORMINHOCO DAS PEDRAS**

.................................................................................................................*Menippe nodifrons*

GOIAMUM

"They are very blue, the color of aniline or light blue. They're larger than the "*goiamun caboclo*.".........................................................................................**GOIAMUM AZULÃO**

.............................................................................................................*Cardisoma guanhumi*

"Bright blue with yellow"............................................................**GOIAMUM CABOCLO**

.............................................................................................................*Cardisoma guanhumi*

SIRI

"*They're blue and large, and live in the mangrove swamp*."................................................................................................................**SIRI AÇÚ**

..........................................................................................................*Callinectes exasperatus*

"*They are long and their shell has very thin teeth. You catch them during the tide. They're meatier and light blue*."..............................................................**SIRI PONTINHA**

...................................................................................................................*Callinectes danae*

"*They are the biggest. Smell like shit when you cook them, they have one reddish claw, they are dark and rusty-colored. They live in fresh water and in pens*."..................................................................................................... **SIRI CAGÃO (M)**

...............................................................................................................*Callinectes bocourti*

*"Fat, somewhat reddish. Live in freshwater."....................................***SIRI NEMA (F)**

...............................................................................................................*Callinectes bocourti*

*"Completely red, the color of malagueta peppers. They burn, just like hot peppers."They live on the rocks and on the edges of the tide which has some stones.".......................................................................................***SIRI PIMENTA**

...............................................................................................................*Callinectes larvatus*

"*They are brownish with white spots, the same color as the guiné plant. Live among the rocks*."..................................................................... **SIRI PINTADO/SIRI DAS PEDRAS**

................................................................................................................*Arenaeus cribrarius*

Legend: M – male; F – female.
